# Supplementary material for: Targeted inhibition of SCFSKP2 confers anti-tumor activities resulting in a survival benefit in osteosarcoma
Source: Oncogene. 2024 Feb 14;43(13):962–75. doi: 10.1038/s41388-024-02942-4 (PMC10959747; doi:10.1038/s41388-024-02942-4)
Supplement: Supplementary file 1 — supp_file [file 41388_2024_2942_MOESM1_ESM.docx]

**Supplementary Materials**

**A targeted inhibition of SCF^SKP2^ has anti-tumor activities that result in a survival benefit in osteosarcoma**

Jichuan Wang^1,2#^, Alexander Ferrena^1,3#^, Ranxin Zhang^1#^, Swapnil Singh^1^, Valentina Viscarret^1^, Waleed Al-Harden^1^, Osama Aldahamsheh^1^, Hasibagan Borjihan^1^, Amit Singla^1^, Simon Yaguare^1^, Janet Tingling^1^, Xiaolin Zi^4^, Yungtai Lo^5^, Richard Gorlick^6^, Edward L. Schwartz^7^, Hongling Zhao^8^, Rui Yang^1^, David S. Geller^1^, Deyou Zheng^9*^ and Bang H. Hoang^1*^

^1^Department of Orthopedic Surgery, Montefiore Medical Center, Albert Einstein College of Medicine, Bronx, NY.

^2^Musculoskleletal Tumor Center, Beijing Key Laboratory for Musculoskeletal Tumors, Peking University People's Hospital, Beijing, China

^3^Institute for Clinical and Translational Research, Departments of Genetics, Albert Einstein College of Medicine, Bronx, NY.

^4^Department of Urology, University of California, Irvine Medical Center, Orange, CA.

^5^Department of Epidemiology & Population Health, Albert Einstein College of Medicine, Bronx, NY.

^6^Division of Pediatrics, University of Texas MD Anderson Cancer Center, Houston, TX.

^7^ Departments of Oncology, Molecular Pharmacology, and Medicine, Albert Einstein College of Medicine, Bronx, NY.

^8^ Department of Developmental and Molecular Biology, Albert Einstein College of Medicine, Bronx, NY.

^9^ Departments of Genetics, Neurology and Neuroscience. Albert Einstein College of Medicine, Bronx, NY.

^#^ Authors contributed equally.

^*^Corresponding author. Bang H. Hoang, Department of Orthopedic Surgery, Montefiore Medical Center, Albert Einstein College of Medicine, Bronx, NY.

E-mail address: bahoang@montefiore.org.

Deyou Zheng, Departments of Genetics, Neurology and Neuroscience. Albert Einstein College of Medicine, Bronx, NY.

E-mail address: deyou.zheng@einsteinmed.edu

**Supplementary Tables**

**Table S1** Genotyping primers used for identification of Skp2 knockout transgenic mice

**Table S2** qPCR primers used in this study

**Table S3** Table showing results of survival analysis of the Reactome Apoptosis gene signature in the NCI TARGET OS cohort

**Supplementary Figures**

**Figure S1**

Genotyping results using agarose gel electrophoresis. (A) Genotyping results of *Trp53* wild type(*p53^(+/+)^* )(288bp), p53-lox heterozygotes (*p53^(lox/+)^* ) (288bp and 370bp) and p53-lox homozygotes (*p53^(lox/lox)^* ) (370bp). (B) Genotyping results of Osterix1-Cre (*Osx-Cre(+)*) (198bp) and coresponding internal positive control（*Osx-Cre(+)* Ctrl) (253bp). (C) Genotyping results of *Rb1* wild type (*Rb1 ^(+/+)^* )(250bp), Rb1-lox heterozygotes (*Rb1 ^(lox/+)^* ) (250bp and 310bp) and Rb1-lox homozygotes (*Rb1 ^(lox/lox)^ )* (310bp). (D) Genotyping results of recombined *Trp53* and *Rb1* after deletion mediated by Osterix-Cre. (～500 bp). (E) Genotyping results of wild type *p27* (250bp), *p27 T187A* heterozygotes (*p27^(T187A/+)^*) (250bp and 284bp) and T187A homozygotes *(p27^(T187A/T187A)^* ) (284bp). (F) Genotyping results of wild type *Skp2* (*Skp2^(wt/wt)^*) (500bp only) and *Skp2 knockout* homozygotes *(Skp2^(-/-)^* ) (430bp only).

**Figure S2**

Sashimi plots showing splicing RNA-seq reads mapped to the *Trp53* (A), *RB1* (B) and Skp2 loci(C). The numbers above the arch lines indicate the numbers of reads for individual splicing patterns. (C) Summary of RNA-seq reads mapped to the T187A mutation sites in the DKO (top) and DKOAA (bottom), confirming mutation in DKOAA samples.

**Figure S3**

Kaplan-Meier survival analysis comparing the DKO, DKOAA and TKO cohorts of mice undergoing tumorigenesis exclude maxillofacial tumor. P-value is by a log-rank test and indicated in the figure.

**Figure S4**

Graph showing the tumor anatomical distribution of each genotype.

**Figure S5**

Mouse body weight during daily treatment of 40 days with either C1 or Pevonedistat as indicated. Statistical significance is indicated by *P < .05,**P < .01, ***P < .001. Column: mean; Error bars are SEM.

**Table S1 Genotyping primers used for identification of Skp2 knockout transgenic mice**

| Wildtype allele | KN3 | 5′- AGAGTGGAAGAACCCAGGCAGGAC-3’ |
| --- | --- | --- |
|  | KN4 | 5′- CCCGTGGAGGGAAAAAGAGGGACG-3’ |
| Knockout allele | KN13 | 5′- GCATCGCCTTCTATCGCCTTCTTG-3’ |
|  | KN38 | 5′- TTCCCACCCCCACATCCAGTCATT-3’ |

**Table S2 qPCR primers used in this study**

| CDKN1B | sense | 5′-GCGGTGCCTTTAATTGGGTCT |
| --- | --- | --- |
|  | antisense | 5′-GGCTTCTTGGGCGTCTGC T |
| p73 | sense | 5′-AACGCCGAGCATCAATCC |
|  | antisense | 5′-AGCCCAGACTCTGAGCACTT |
| Skp2 | sense | 5′-AGCAGCCGCTGGGTGAAAGC |
|  | antisense | 5′-ATCACTGAGTTCGACAGGTCCAT |
| E2f1 | sense | 5′-TCACTAAATCTGACCACCAAACG |
|  | antisense | 5′-TTGGACTTCTTGGCAATGAGC |
| Bbc3(Puma) | sense | 5′-GGTCCAGACTGTGAATCCTGTG |
|  | antisense | 5′-TCCTCCCTCTTCTGAGACTTCC |
| Bid | sense | 5′-ACGGAATGCAAAGAACAACTC |
|  | antisense | 5′-CAACGCTTGAGGATACAGTGAG |
| Bcl2l11(Bim) | sense | 5′-ATCTTGTTGGGCTTACTTGTG |
|  | antisense | 5′-GTCCTGCCTGGTCTTGAAAT |
| Casp3 | sense | 5′-CGCGCACAAGCTAGAATTTATG |
|  | antisense | 5′-GGACACAATACACGGGATCTG |
| Prominin1 | sense | 5′- TCTGCTGACATTTGCCTCTAC |
|  | antisense | 5′- GCTGGTGGATGGCTCTTATATT |
| ALDH1A1 | sense | 5′- GAGAGTGGGAAGAAAGAAGGAG |
|  | antisense | 5′- CTCATCAGTCACGTTGGAGAA |
| ALDH2 | sense | 5′- CATCTTGGTACCTGGGATCTTG |
|  | antisense | 5′- TGTAGCTGCAGCCAAGAATAG |
| ALDH7A1 | sense | 5′- CTCAGTACCACCACAACAAAGA |
|  | antisense | 5′- CAAGAACGAAGGTCTGTCTACC |
| CD117(Kit) | sense | 5′- AGGAGAACTGAGGCTGTTTG |
|  | antisense | 5′- TAACTTGTGCTCCCTGCTATG |
| GAPDH | sense | 5′- GGTTGTCTCCTGCGACTTCA |
|  | antisense | 5′-GGTGGTCCAGGGTTTCTTAC |

**Table S3.**


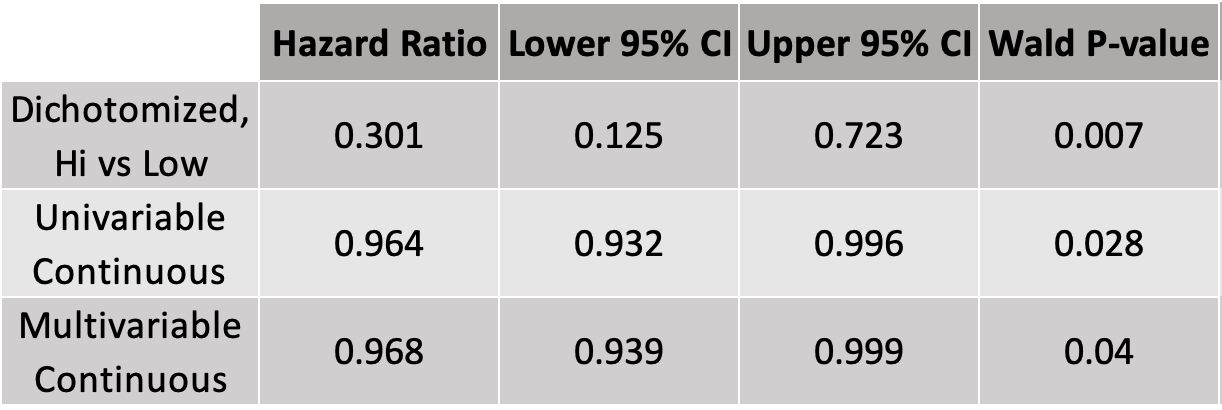


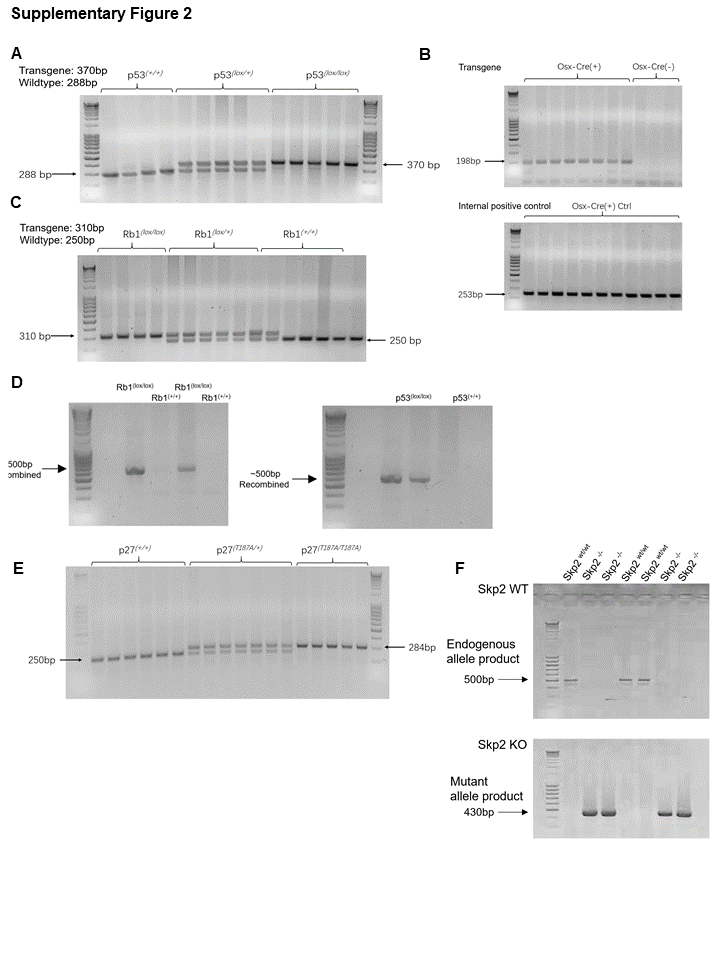
**Figure S1**


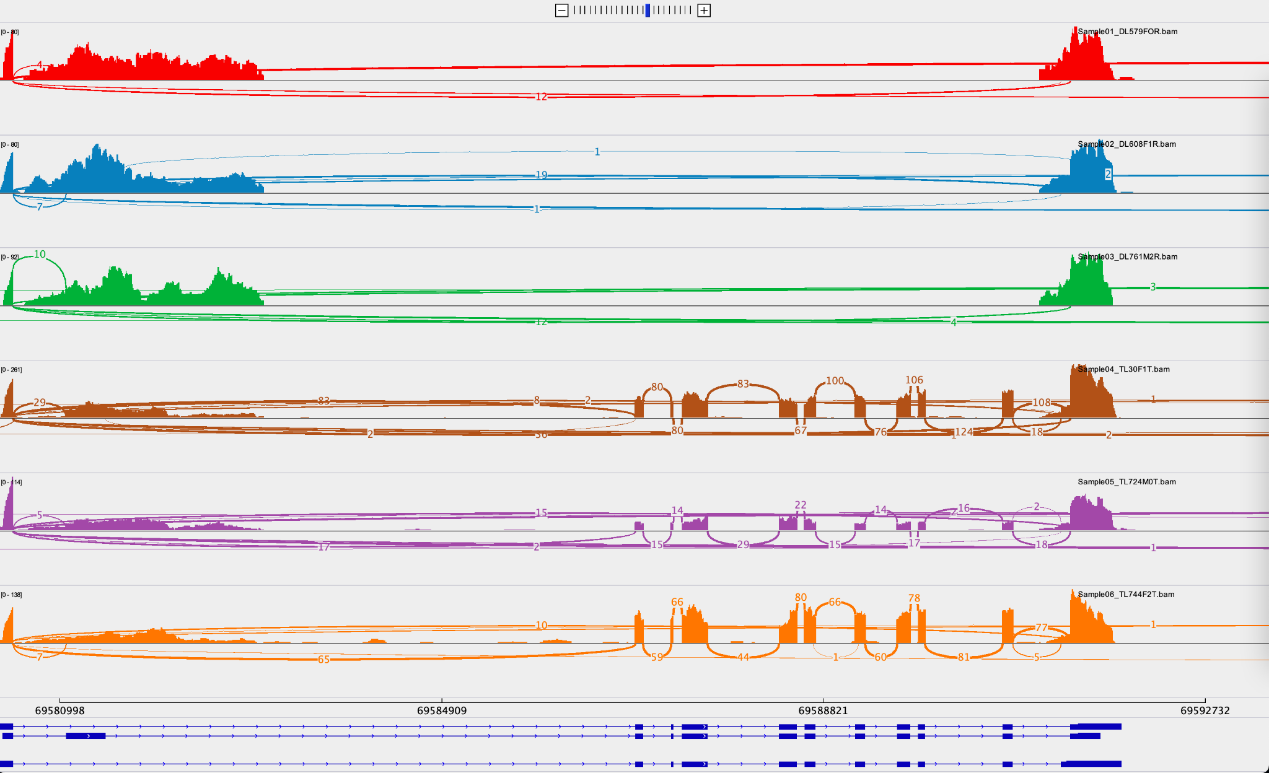
**Figure S2**

A) Splicing reads at exons 2-10 excision of Trp53, DKO (up) and TKO (down)

B) Splicing reads at exons 3 excision of RB1, DKO (up) and TKO (down).


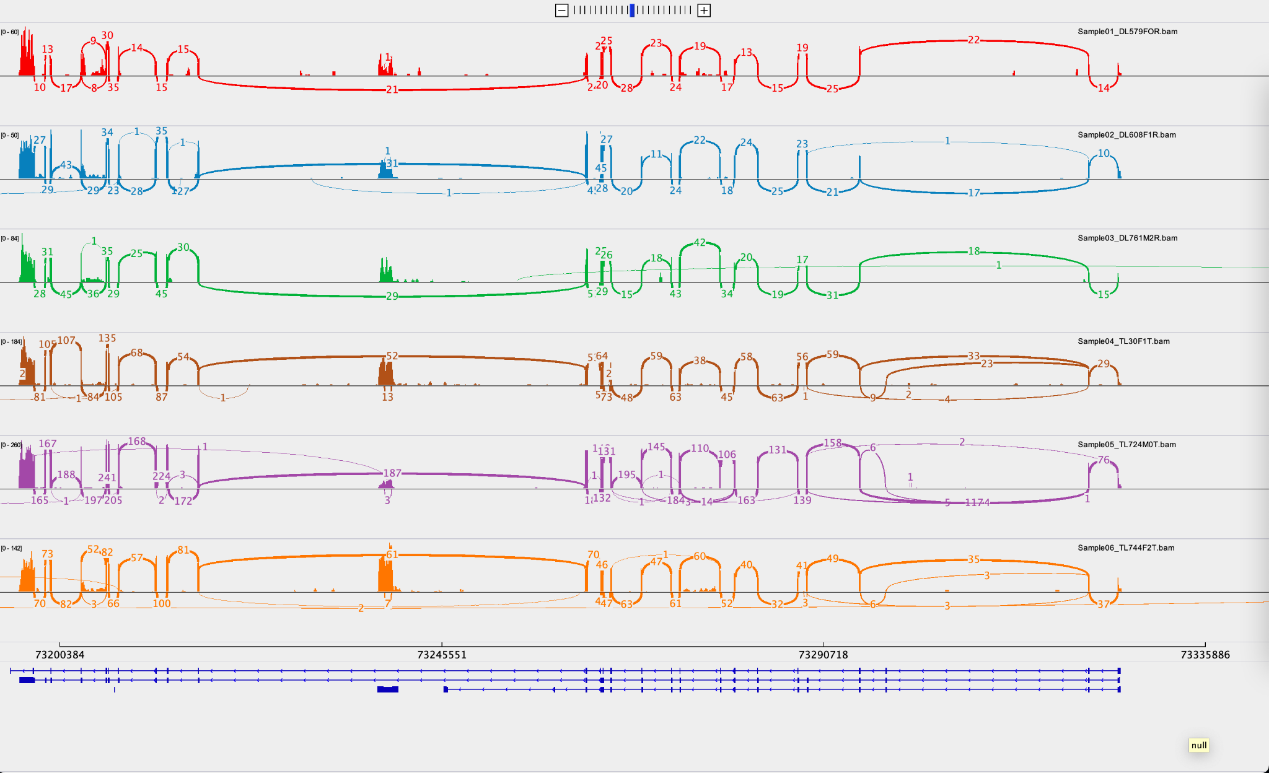


C) Splicing reads showing deletion of Skp2, DKO (up), and TKO (down).


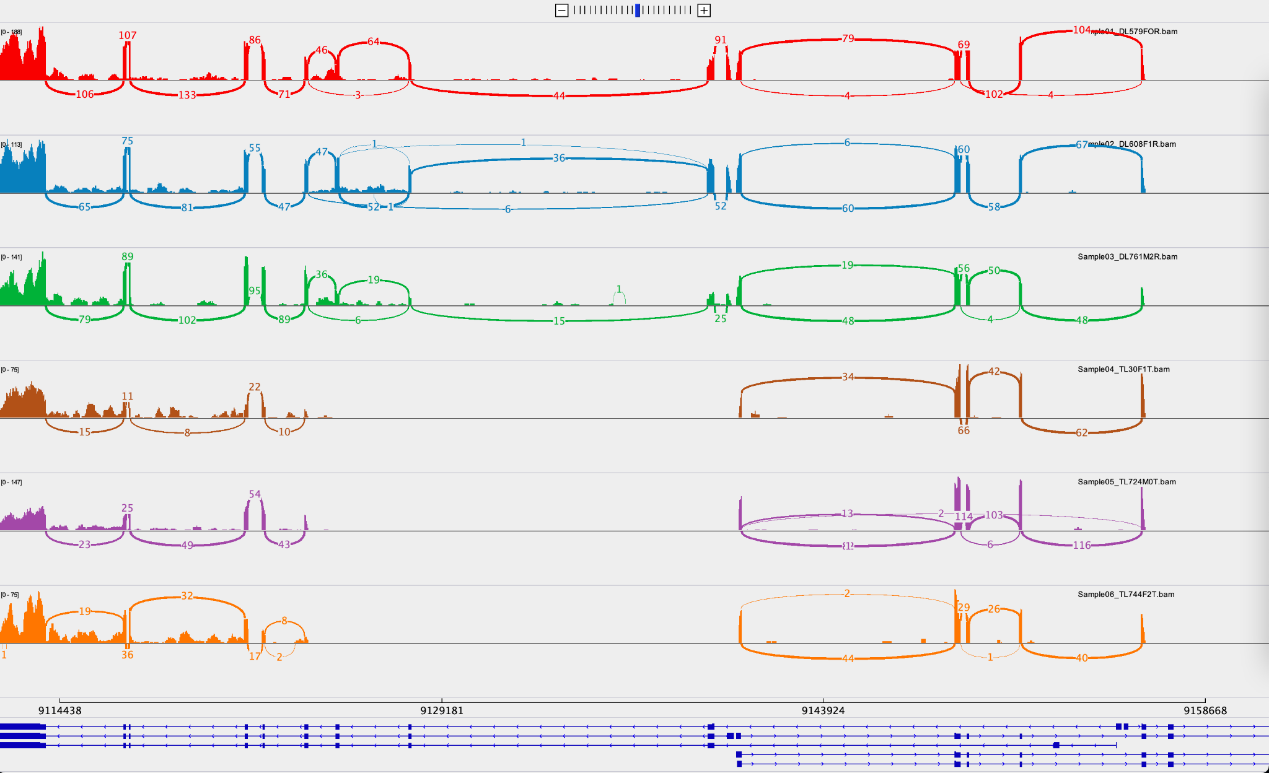


**Figure S3**


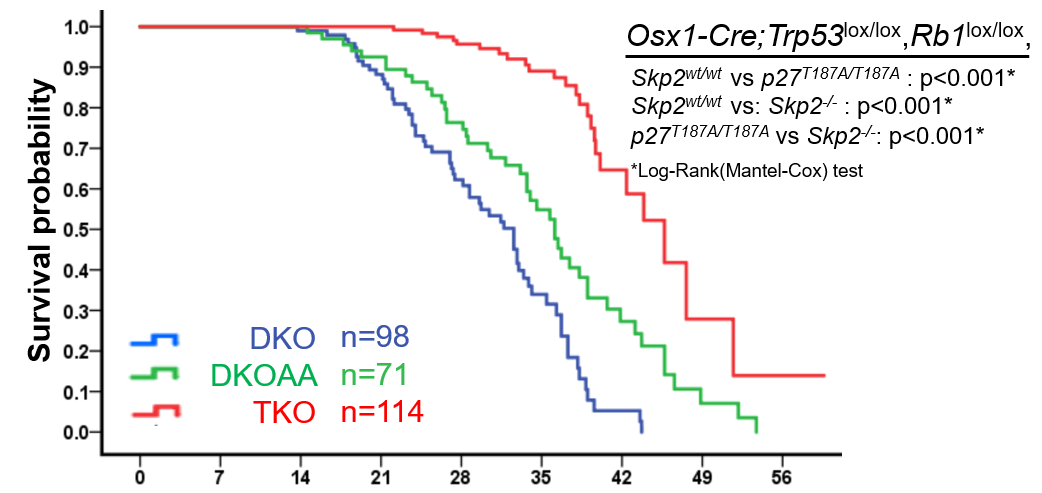


**Figure S4**


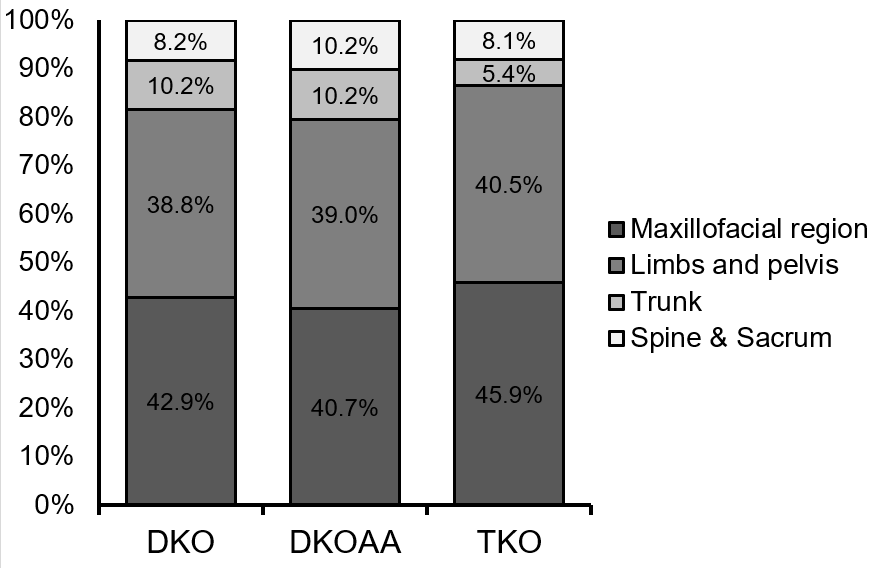

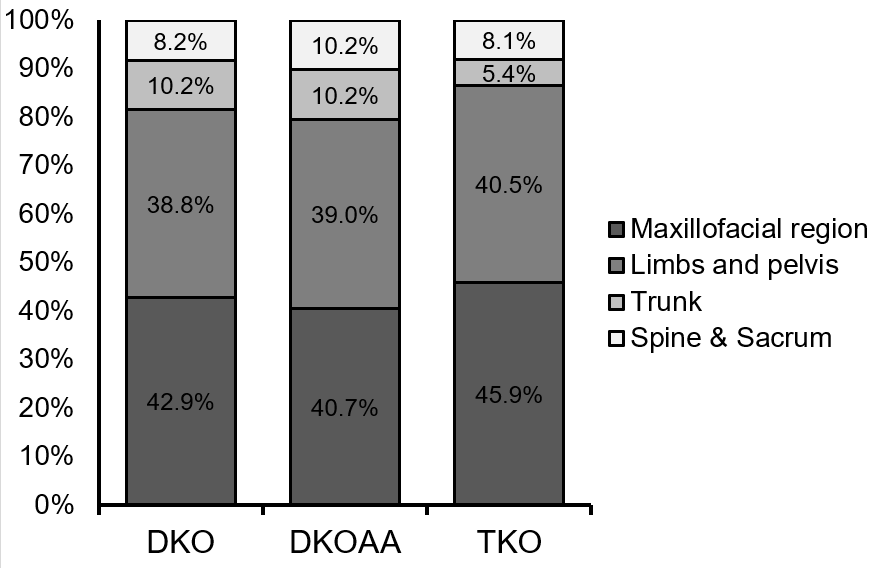


**Figure S5**

**
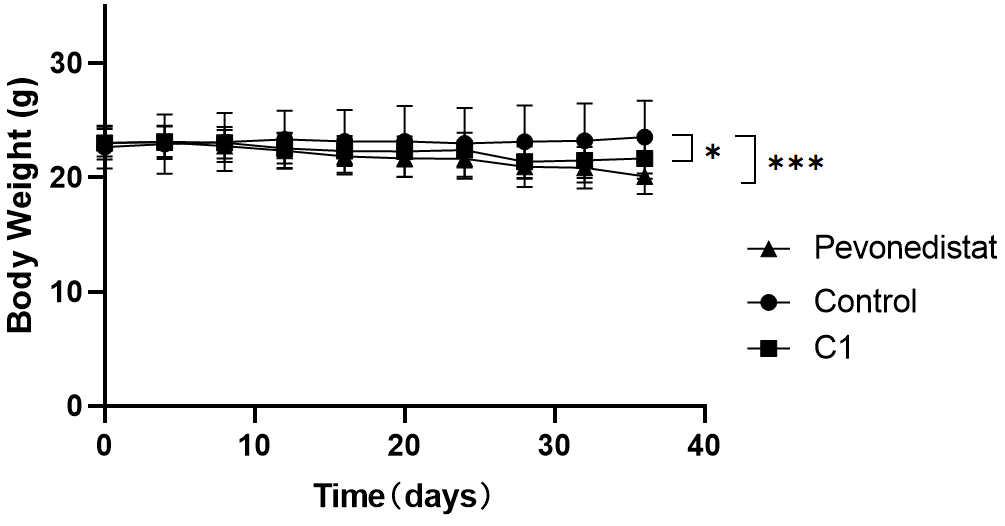
**
